# Incidence of Colorectal Cancer After Endoscopic Mucosal Resection: A Multicenter Electronic Health Record Cohort Study

**DOI:** 10.1007/s12029-026-01634-4

**Published:** 2026-11-07

**Authors:** Mohamed H. Eldesouki, Khaled Elfert, EzzElDien A. Ibrahim, Mohammed Y. Youssef, Aasma Shaukat, Shyam Thakkar

**Affiliations:** 1 Department of Internal Medicine, New York Medical College at Saint Michael’s Medical Center, Newark, NJ USA; 2 Division of Gastroenterology and Hepatology, West Virginia University, Morgantown, WV USA; 3 Department of Medicine, University of Missouri-Columbia, Columbia, MO USA; 4 Department of Internal Medicine, Hunt Regional Medical Center, Greenville, TX USA; 5 Division of Gastroenterology and Hepatology, NYU Grossman School of Medicine, NYU Langone Health, New York, NY USA

**Keywords:** Endoscopic mucosal resection, Colorectal cancer, Incidence, Surveillance, TriNetX, Real-world data

## Abstract

**Background:**

Endoscopic mucosal resection (EMR) is widely used for large and complex colorectal lesions and offers a less invasive alternative to surgery. Although adenoma recurrence after EMR has been described in procedural cohorts, real-world estimates of subsequent colorectal cancer (CRC) diagnoses after EMR across multicenter electronic health record (EHR) systems remain limited.

**Methods:**

We conducted a retrospective cohort study using the TriNetX US Collaborative Network. Patients aged 40 years or older who underwent colonoscopy or sigmoidoscopy with EMR between January 1, 2016 and January 1, 2017 were included. Patients with Crohn’s disease, ulcerative colitis, familial adenomatous polyposis, or CRC recorded on or before the index EMR date were excluded. The primary outcome was incident CRC, defined by ICD-10-CM codes C18, C19, and C20, assessed from January 1, 2018 through December 31, 2021 after a one-year washout period. Incidence rates are reported per 1,000 person-years with exact Poisson 95% confidence intervals (CIs). A sensitivity analysis was restricted to patients aged 50–75 years at index EMR.

**Results:**

Among 3,081 eligible patients, the mean age was 62.9 ± 8.89 years and 52% were female; 2,735 were at risk at the start of follow-up. Twenty-two incident CRC diagnoses occurred over 10,187.8 person-years, an incidence of 2.16 per 1,000 person-years (95% CI, 1.35–3.27). Each calendar year contained 10 or fewer events, so annual rates could not be estimated. Incidence was 2.54 (95% CI, 1.31–4.44) in males and 1.83 (95% CI, 0.88–3.36) in females. In the 50-75-year sensitivity cohort, 19 events occurred over 8,894.8 person-years (2.14 per 1,000 person-years; 95% CI, 1.29–3.34). During years 1–5 after index EMR, repeat EMR occurred in 119 patients (3.9%), colorectal surgery in 28 (0.9%), and subsequent colorectal polyp diagnoses in 1,321 (42.9%); no ESD procedures were identified.

**Conclusion:**

CRC diagnoses after EMR were uncommon in this multicenter EHR cohort, occurring at about 2 per 1,000 person-years. Event counts were too small to estimate annual trends or to compare incidence reliably across race and ethnicity groups. Studies linking EHR data to endoscopy and pathology reports are needed to identify preventable cancers after EMR.

## Introduction

Colorectal cancer (CRC) remains a major cause of cancer-related morbidity and mortality in the United States. Although screening and treatment have improved, CRC burden continues to vary across racial, ethnic, sex, and socioeconomic groups [1, 2]. Colonoscopy is central to CRC prevention because detection and removal of precursor lesions reduce subsequent cancer risk [3].

Endoscopic mucosal resection (EMR) has become an established approach for large, flat, sessile, or laterally spreading colorectal lesions that are not readily removed by standard polypectomy [4]. Compared with segmental surgery for benign or superficially invasive lesions, EMR can preserve the colon and avoid perioperative morbidity when performed in appropriate lesions by experienced endoscopists. However, EMR outcomes depend on lesion size, morphology, histology, location, en bloc versus piecemeal technique, completeness of excision, margin treatment, and surveillance [5–7].

Recurrence after EMR is well described in procedural cohorts. In a meta-analysis of nonpedunculated colorectal lesions, local recurrence after EMR occurred in approximately 15% of cases overall and was more common after piecemeal resection than en bloc resection [8]. More recent data suggest that systematic margin treatment and careful surveillance can substantially reduce recurrence [9, 10]. Consensus recommendations for follow-up after colonoscopy and polypectomy also emphasize early surveillance after piecemeal resection of large colorectal lesions [11].

A later CRC diagnosis after colonoscopy or endoscopic resection can represent several different processes: a missed synchronous lesion, incompletely resected neoplasia, local recurrence at the resection site, delayed surveillance, or a new metachronous cancer [12, 13]. The World Endoscopy Organization has standardized terminology and review methods for post-colonoscopy CRC, and contemporary meta-analytic estimates suggest that post-colonoscopy CRC remains an important quality metric [13, 14]. However, most large datasets cannot distinguish the anatomic and mechanistic pathways that lead from EMR to later CRC. This distinction is particularly important in EHR-based studies, where diagnoses are observed through structured codes rather than endoscopy reports, pathology reports, and lesion maps.

Large population cohorts have characterized CRC risk after negative colonoscopy [15], but data specifically focused on CRC diagnoses after colorectal EMR in diverse real-world populations remain limited. Surveillance after complex polyp resection is vulnerable to access barriers, travel distance, language, insurance continuity, and other social determinants of health [16–19]. Current US screening recommendations also provide important context for interpreting age-restricted analyses and average-risk screening populations [20]. These factors may influence whether a patient returns for surveillance and whether a later CRC diagnosis is captured in a participating EHR system.

We used the TriNetX US Collaborative Network to estimate observed CRC diagnoses after EMR among patients aged 40 years or older. We also described sex-, race-, and ethnicity-stratified incidence, a 50-75-year sensitivity cohort, and selected follow-up events after EMR. Because the data source is EHR-based and aggregate, the purpose of the analysis was descriptive rather than mechanistic.

## Methods

### Study Design and Data Source

We conducted a retrospective cohort study using the TriNetX US Collaborative Network, a federated real-world data platform that aggregates de-identified EHR data from participating healthcare organizations. The network used for the final analyses included 66 healthcare organizations. TriNetX captures structured demographic, diagnosis, procedure, medication, and laboratory data using standardized terminologies within a federated architecture that does not transfer raw patient-level records to investigators [21]. Published methodological reviews of TriNetX describe its use for real-world research as well as the need to interpret aggregate EHR output within the constraints of coding quality, data harmonization, and observability [22].

Cohorts were constructed within the TriNetX query interface using ICD-10-CM diagnosis codes, Current Procedural Terminology (CPT) procedure codes, demographic fields, and Boolean logic. Because only de-identified aggregate data were used, the analysis did not involve direct patient contact and was considered exempt from institutional review board review under applicable policies for de-identified data. This approach is consistent with published guidance on responsible use of standardized, de-identified EHR data for research [23].

### Cohort Definition

Eligible patients were aged 40 years or older at the time of index EMR and underwent colonoscopy or sigmoidoscopy with EMR between January 1, 2016 and January 1, 2017. EMR was identified using CPT codes 44403 (colonoscopy through stoma with EMR), 45390 (colonoscopy, flexible, with EMR), and 45349 (sigmoidoscopy, flexible, with EMR). The index period was selected to allow follow-up through December 31, 2021 and to avoid the ICD-9 to ICD-10 transition period.

Exclusions included Crohn’s disease (K50), ulcerative colitis (K51), familial adenomatous polyposis (D13.91), and CRC (C18, C19, or C20) recorded on or before the index EMR date.

### Outcome Definition and Follow-up

Subsequent CRC diagnosis was the primary outcome and was defined using ICD-10-CM codes C18 (malignant neoplasm of colon), C19 (malignant neoplasm of rectosigmoid junction), or C20 (malignant neoplasm of rectum). Outcomes were assessed from January 1, 2018 through December 31, 2021. A one-year washout period after the 2016–2017 EMR index window was used to reduce capture of synchronous, prevalent, or peri-procedural cancers.

Lesion-level anatomic linkage is not available in TriNetX; therefore, later CRC diagnoses could not be classified as residual or recurrent neoplasia at the index EMR site versus metachronous CRC elsewhere in the colorectum.

### Incidence Rate Calculation

For each time window, TriNetX reports the incidence proportion (incident cases divided by patients at risk) and the incidence rate (incident cases divided by person-days at risk). Patients with a CRC diagnosis recorded at any time before the start of a window were classified as prevalent and excluded from the at-risk population for that window. Because both measures are ratios of whole-number counts, we recovered the number of incident cases and patients at risk as the integer pair consistent with the reported incidence proportion, and person-time as incident cases divided by the incidence rate. Person-days were converted to person-years by dividing by 365.25. Incidence rates are reported per 1,000 person-years with exact Poisson 95% CIs.

To protect patient privacy, TriNetX reports any count between 1 and 10 as 10. A reported count of 10 therefore represents 1 to 10 true events. Where the true count of a masked stratum could be obtained exactly by subtraction from unmasked totals, the exact count is reported; otherwise, the count is reported as a range and no rate is given. The combined 2018–2021 window was the primary analysis. Every single-year window contained a masked count, so annual incidence rates were not estimable. Annual person-time is reported to show that it sums to the combined total.

In the TriNetX output, person-time was not truncated at a patient’s last recorded encounter. Mean person-time per at-risk patient was 3.72 of a possible 4.00 years in the combined window and 0.96 to 0.99 of a possible 1.00 year in each calendar-year window. Patients who left participating healthcare organizations therefore continued to contribute person-time, although a CRC diagnosed elsewhere could not be recorded.

### Subgroup and Sensitivity Analyses

Baseline characteristics were summarized for the final cohort. Race and ethnicity were obtained from structured EHR demographic fields, and unknown categories were retained rather than excluded. Incidence was stratified descriptively by sex, race, and ethnicity. These subgroup analyses were exploratory because the number of CRC events was small, several strata had masked counts, and TriNetX aggregate output did not allow multivariable adjustment for surveillance adherence, lesion characteristics, insurance continuity, or socioeconomic factors.

A sensitivity analysis restricted the cohort to patients aged 50–75 years at the time of index EMR. This range was selected because CRC screening and post-polypectomy surveillance are most clinically interpretable in this age range, while very elderly patients may have competing comorbidity and variable surveillance decisions [20]. The same EMR codes, exclusion criteria, outcome definition, and follow-up window were applied.

### Exploratory Follow-up Events

To contextualize post-EMR care patterns, we described selected events occurring from 365 to 1,825 days after index EMR: repeat EMR, endoscopic submucosal dissection (ESD), colorectal surgery, and subsequent colorectal polyp diagnoses. These events were not used to define the primary outcome and could not be anatomically linked to the index EMR site.

### Statistical Analysis

Continuous variables are reported as mean ± standard deviation, and categorical variables are reported as n (%). Incidence rates are presented with exact Poisson 95% CIs calculated from the number of events and person-years. Estimates are descriptive. No causal modeling was performed because the database did not provide patient-level procedural details, surveillance adherence, lesion-level linkage, or complete information on care received outside participating healthcare organizations. Cohort construction and incidence analyses were performed within the TriNetX platform.

## Results

### Patient Characteristics

Among 3,081 patients aged 40 years or older at index EMR, mean age was 62.9 ± 8.89 years; 1,599 (52%) were female and 1,482 (48%) were male (Table 1). Unknown race and ethnicity were retained to show the extent of missing demographic documentation.

**Table 1:** Baseline characteristics of patients undergoing endoscopic mucosal resection

Characteristic	Overall cohort, *N* = 3,081
Age at index, years, mean ± SD	62.9 ± 8.89
Female sex	1,599 (52%)
Male sex	1,482 (48%)
Ethnicity	
Not Hispanic or Latino	2,636 (86%)
Hispanic or Latino	76 (2%)
Unknown ethnicity	369 (12%)
Race	
White	2,344 (76%)
Black or African American	351 (11%)
Asian	155 (5%)
Unknown race	152 (5%)
Native Hawaiian or Other Pacific Islander	45 (1%)
Other race	24 (1%)
American Indian or Alaska Native	10 (< 1%)
Relevant baseline factors	
Type 2 diabetes mellitus	485 (16%)
Overweight or obesity	449 (15%)
Family history of malignant neoplasm of digestive organs	202 (7%)
Personal history of nicotine dependence	315 (10%)

Values are presented as n (%) unless otherwise specified. *SD *standard deviation. Diagnoses are based on structured EHR fields and may be affected by undercoding

### Observed CRC Incidence

At the start of the 2018–2021 window, 2,735 patients were at risk; patients with a CRC diagnosis recorded before January 1, 2018 were excluded as prevalent. Twenty-two incident CRC diagnoses occurred over 10,187.8 person-years, a combined incidence of 2.16 per 1,000 person-years (95% CI, 1.35–3.27) (Table 2). Annual person-time was 2,693.9, 2,603.6, 2,517.1, and 2,373.3 person-years in 2018, 2019, 2020, and 2021, respectively, and summed to the combined total. Each single-year window contained between 1 and 10 incident events, which TriNetX reports as 10; annual rates were therefore not estimable.

When the cohort was restricted to patients aged 50–75 years at index EMR, 2,664 patients were included, of whom 2,380 were at risk at the start of follow-up. Nineteen incident CRC diagnoses occurred over 8,894.8 person-years (2.14 per 1,000 person-years; 95% CI, 1.29–3.34). As in the primary cohort, each single-year window contained a masked count.

**Table 2:** CRC events, person-time, and incidence after EMR in the primary and sensitivity cohorts

Analysis / window	Patients at risk	CRC events	Person-years	Incidence per 1,000 person-years (95% CI)
Primary cohort (40 years or older), 2018–2021	2,735	22	10,187.8	2.16 (1.35–3.27)
2018	2,735	1–10	2,693.9	NE
2019	2,654	1–10	2,603.6	NE
2020	2,563	1–10	2,517.1	NE
2021	2,462	1–10	2,373.3	NE
Sensitivity cohort (50–75 years), 2018–2021	2,380	19	8,894.8	2.14 (1.29–3.34)
2018	2,380	1–10	2,346.1	NE
2019	2,316	1–10	2,274.2	NE
2020	2,238	1–10	2,199.8	NE
2021	2,151	1–10	2,074.8	NE

CRC events were 10 or fewer in every single-year window. TriNetX reports counts between 1 and 10 as 10 to protect patient privacy, so annual rates were not estimable. Annual person-years sum to the combined total (differences reflect rounding). Rates are per 1,000 person-years with exact Poisson 95% CIs. *CI *confidence interval, *CRC *colorectal cancer, *EMR *endoscopic mucosal resection, *NE *not estimable

### Sex-, Race-, and Ethnicity-Stratified Incidence

**Fig. 1:**
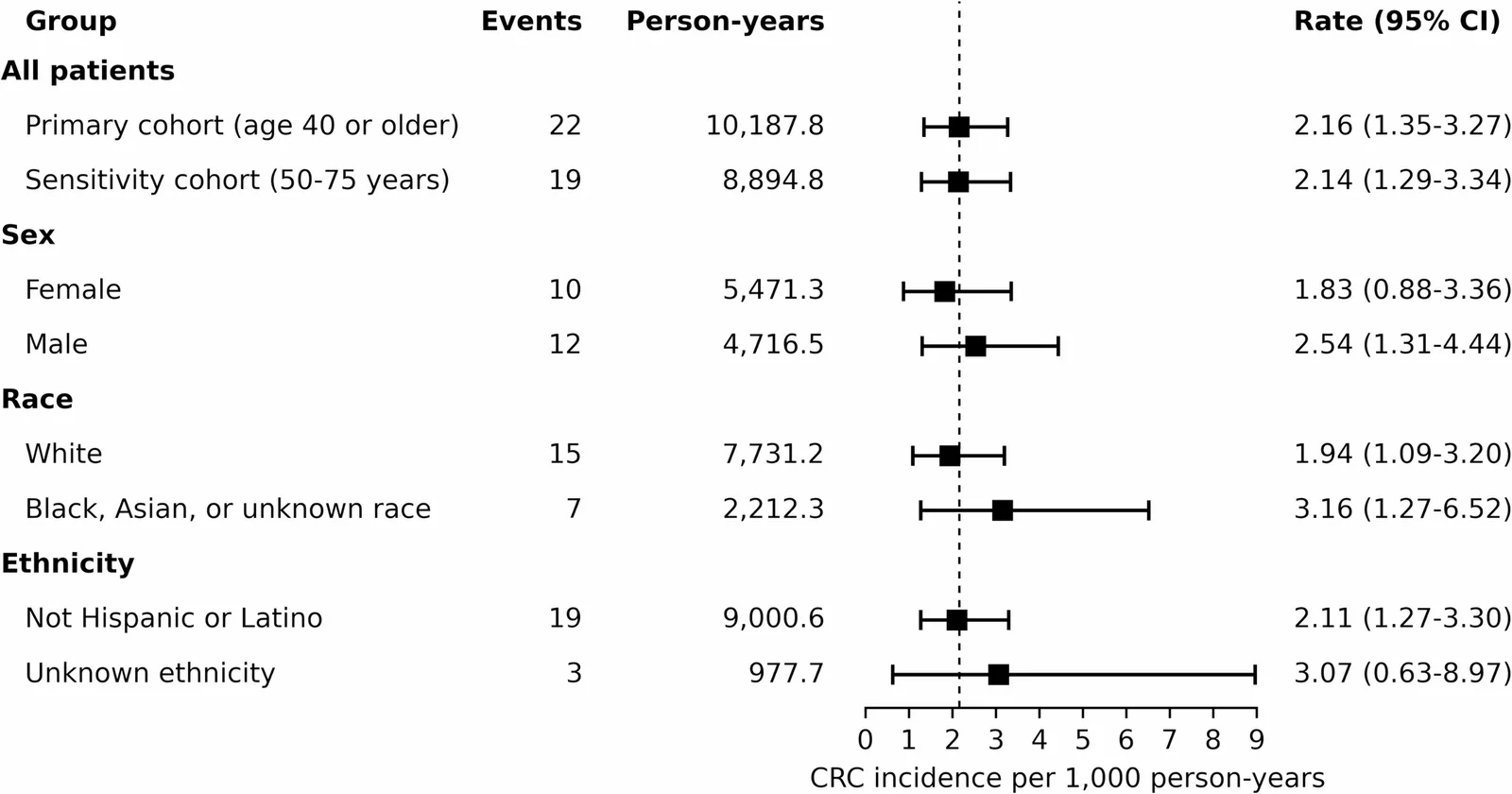
CRC incidence after EMR overall and by sex, race, and ethnicity, 2018–2021. Squares show incidence per 1,000 person-years and horizontal lines show exact Poisson 95% confidence intervals. The dashed line marks the primary cohort estimate (2.16 per 1,000 person-years). Black or African American, Asian, and unknown-race patients are shown combined because their individual counts were masked by TriNetX. CRC, colorectal cancer; EMR, endoscopic mucosal resection; CI, confidence interval

In the primary cohort, incidence was 2.54 per 1,000 person-years (95% CI, 1.31–4.44; 12 events) in males and 1.83 (95% CI, 0.88–3.36; 10 events) in females. White patients had 15 events over 7,731.2 person-years (1.94; 95% CI, 1.09–3.20). Black or African American, Asian, and unknown-race patients together had 7 events over 2,212.3 person-years (3.16; 95% CI, 1.27–6.52); counts within each of these groups were masked and could not be separated. No events occurred among American Indian or Alaska Native, Native Hawaiian or Other Pacific Islander, other-race, or Hispanic or Latino patients. Non-Hispanic patients had 19 events (2.11; 95% CI, 1.27–3.30), and patients with unknown ethnicity had 3 events (3.07; 95% CI, 0.63–8.97) (Table 3; Fig. 1). Confidence intervals overlapped across all sex, race, and ethnicity groups.

**Table 3:** CRC incidence by sex, race, and ethnicity in the primary cohort, 2018–2021

Subgroup	Patients at risk	CRC events	Person-years	Incidence per 1,000 person-years (95% CI)
Sex				
Female	1,447	10*	5,471.3	1.83 (0.88–3.36)
Male	1,288	12	4,716.5	2.54 (1.31–4.44)
Race				
White	2,078	15	7,731.2	1.94 (1.09–3.20)
Black or African American	325	1–5†	1,236.1	NE
Asian	150	1–5†	562.7	NE
Unknown race	116	1–5†	413.5	NE
Black, Asian, or unknown race (combined)	591	7	2,212.3	3.16 (1.27–6.52)
American Indian or Alaska Native, Native Hawaiian or Other Pacific Islander, or other race‡	66	0	NR	0
Ethnicity				
Not Hispanic or Latino	2,390	19	9,000.6	2.11 (1.27–3.30)
Hispanic or Latino	62	0	NR	0
Unknown ethnicity	283	3*	977.7	3.07 (0.63–8.97)

Incidence was similar between males and females and across race and ethnicity groups, with overlapping confidence intervals. * Exact count obtained by subtraction from unmasked totals (females: 22 total minus 12 in males; unknown ethnicity: 22 total minus 19 non-Hispanic and 0 Hispanic). † Reported by TriNetX as 10 (true count 1 to 10). These three groups had 7 events in total (22 minus 15 in White patients), so each had 1 to 5 events. ‡ Patients at risk across these three groups combined. Estimates are unadjusted and based on aggregate TriNetX output; lesion characteristics, EMR quality, surveillance adherence, socioeconomic factors, and care outside participating HCOs were unavailable. Rates are per 1,000 person-years with exact Poisson 95% CIs. *NE *not estimable, *NR *not reported (person-time is not returned when no events occur)

### Exploratory Follow-up Events

**Fig. 2:**
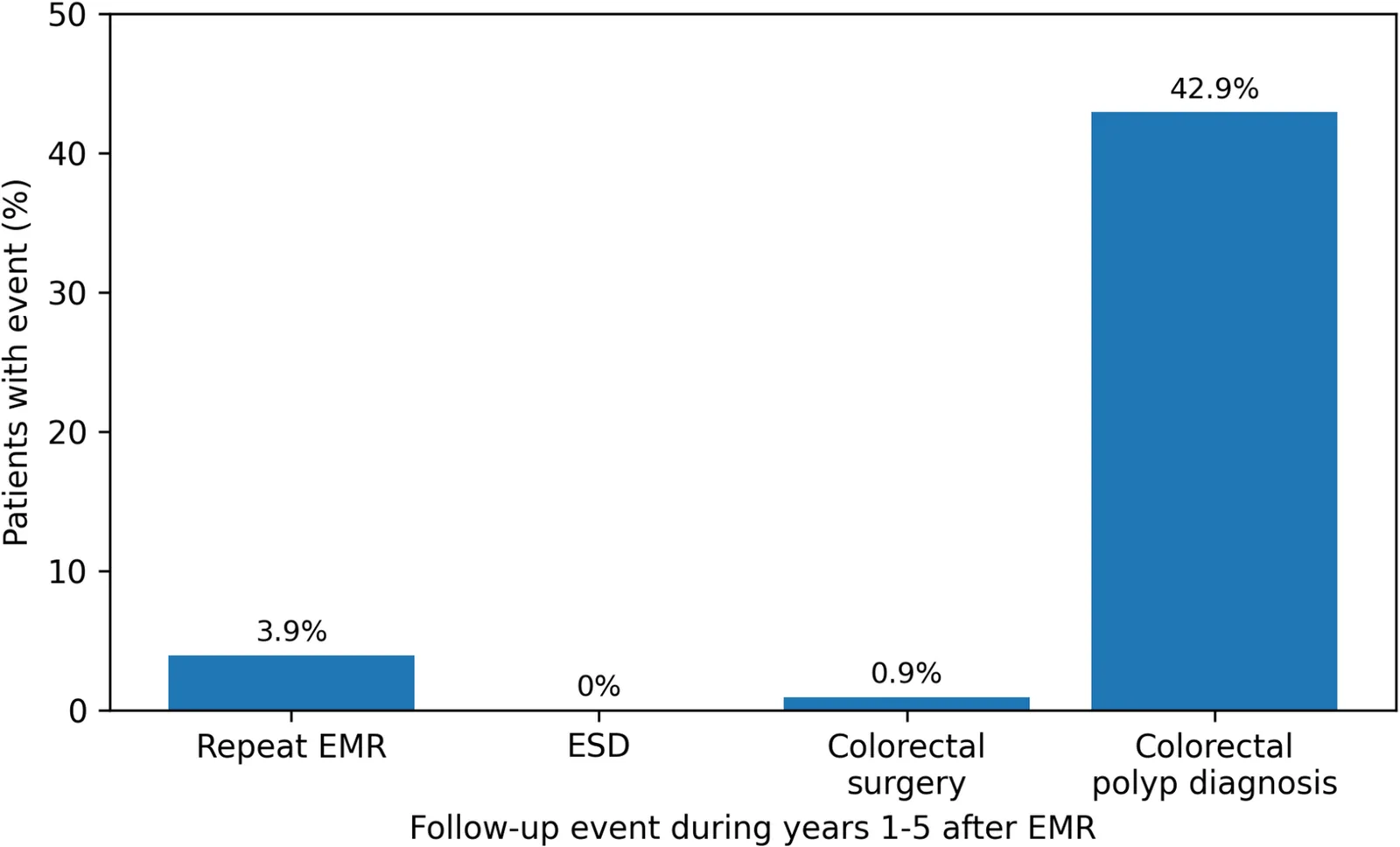
Exploratory follow-up events during years 1–5 after index EMR. Events were assessed from 365 to 1,825 days after the index EMR and could not be anatomically linked to the index lesion. ESD, endoscopic submucosal dissection; EMR, endoscopic mucosal resection

During years 1–5 after index EMR, 119 patients (3.9%) underwent repeat EMR, 28 (0.9%) underwent colorectal surgery, and 1,321 (42.9%) had subsequent colorectal polyp diagnoses; no ESD procedures were identified (Table 4; Fig. 2). These events provide context for post-EMR care patterns but cannot be linked to the index EMR lesion.

**Table 4:** Exploratory follow-up events during years 1–5 after index EMR

Follow-up event	Patients with event, *n*/*N* (%)
Repeat EMR	119/3,081 (3.9%)
Endoscopic submucosal dissection	0/3,081 (0%)
Colorectal surgery	28/3,081 (0.9%)
Subsequent colorectal polyp diagnosis	1,321/3,081 (42.9%)

Outcomes were assessed from 365 to 1,825 days after index EMR. *EMR *endoscopic mucosal resection, *ESD *endoscopic submucosal dissection

## Discussion

In a multicenter EHR-based cohort of patients aged 40 years or older undergoing colorectal EMR, subsequent CRC diagnoses were uncommon but measurable. Twenty-two incident CRC diagnoses occurred over 10,187.8 person-years from 2018 to 2021, an incidence of 2.16 per 1,000 person-years (95% CI, 1.35–3.27), with a nearly identical estimate in the 50-75-year sensitivity cohort. These findings provide a pragmatic real-world benchmark for CRC diagnoses after EMR, while underscoring the limits of administrative and EHR-based outcome ascertainment.

Compared with general population CRC rates for similar age groups, the observed post-EMR rate is higher, which is biologically and clinically plausible because patients selected for EMR have already demonstrated advanced or complex colorectal neoplasia [1, 2]. At the same time, the estimate is much lower than local recurrence rates reported in procedural EMR cohorts [6–10]. This difference is expected because the present outcome captures EHR-coded CRC diagnoses rather than endoscopic recurrence of adenoma or dysplasia at the resection site.

Annual incidence could not be estimated. Each single-year window contained 10 or fewer events, which TriNetX reports as 10. Rates calculated from these masked counts would overstate incidence and would create an artificial upward trend, because the masked numerator stays fixed while person-time declines each year. Larger cohorts or longer follow-up are needed to assess whether CRC risk after EMR changes over time.

Incidence was higher in males than females (2.54 vs. 1.83 per 1,000 person-years), which is directionally consistent with the known male predominance in CRC risk [1]. The confidence intervals overlapped widely, and no patient-level adjustment was possible, so this difference should not be considered established.

Disparities in CRC screening, interval cancer, surveillance adherence, and access to follow-up care have been reported in prior studies [2, 16–19]. In this cohort, Black, Asian, and unknown-race patients together had a higher point estimate of incidence than White patients (3.16 vs. 1.94 per 1,000 person-years). However, the confidence intervals overlapped and the comparison rested on 7 events. Counts within individual minority groups were masked and could not be estimated. This study therefore cannot confirm or exclude racial or ethnic differences in CRC after EMR. Larger datasets with complete demographic capture and surveillance data are needed to address this question.

Restricting the analysis to patients aged 50–75 years was important for interpretability. Patients undergoing EMR before age 50 may represent a selected group undergoing diagnostic colonoscopy or high-risk surveillance rather than average-risk screening, whereas older patients may have competing comorbidities and variable surveillance decisions [20]. The similar incidence estimate in the 50-75-year sensitivity analysis supports the robustness of the overall descriptive finding.

Exploratory follow-up events add useful context. Repeat EMR and colorectal surgery were uncommon, while subsequent colorectal polyp diagnoses were frequent. The high polyp diagnosis rate suggests ongoing surveillance or repeat colonoscopy in many patients, but TriNetX codes cannot determine whether later polyps represented residual lesion, recurrent adenoma, new metachronous lesions, or coding carry-forward from prior findings.

Important strengths include the use of a large multicenter EHR network, an index EMR window with exclusion of prior CRC and selected high-risk conditions, transparent reporting of event counts, person-time, and confidence intervals, retention of unknown race and ethnicity categories, and inclusion of an age-restricted sensitivity analysis. The analysis also provides practical context on selected follow-up procedures and diagnoses after EMR.

Several limitations should be acknowledged. First, TriNetX relies on structured ICD-10-CM and CPT codes for cohort identification and outcome ascertainment, so miscoding, undercoding, and coding differences across institutions are possible; this limitation is inherent to large federated EHR networks and has been described in prior TriNetX methodology literature [21, 22]. Second, responsible use of de-identified aggregate EHR data requires caution when interpreting missingness, observability, and cross-site data harmonization [23]. Third, the platform lacks lesion-level and procedure-level variables, including lesion size, location, morphology, histology, fibrosis, en bloc versus piecemeal resection, margin thermal ablation, completeness of resection, complications, and endoscopist experience. These factors are central determinants of residual or recurrent neoplasia after EMR and could not be adjusted for [5–11]. Fourth, subsequent CRC diagnoses could not be linked anatomically to the index EMR site, preventing differentiation between local residual or recurrent neoplasia and metachronous CRC. Fifth, surveillance adherence, insurance continuity, socioeconomic status, educational attainment, provider adenoma detection rate, and care received outside participating HCOs could not be measured. Sixth, race and ethnicity were based on structured EHR fields and included unknown categories; missing demographic data may bias subgroup estimates. Seventh, the number of CRC events was small (22 in the primary cohort), limiting precision and precluding adjusted subgroup analysis. TriNetX reports counts of 1 to 10 as 10, which prevented estimation of annual rates and of rates within several race and ethnicity groups. Finally, because TriNetX is a federated EHR network rather than a closed claims database, disenrollment, migration to non-participating systems, and care received outside the network could not be measured. Person-time was not truncated at the last recorded encounter, so patients who left participating organizations continued to contribute person-time. A CRC diagnosed elsewhere would be missed, which would bias incidence downward, particularly in later follow-up years.

## Conclusion

Among patients aged 40 years or older undergoing EMR in a multicenter EHR network, 22 incident CRC diagnoses occurred over 10,187.8 person-years, an incidence of 2.16 per 1,000 person-years (95% CI, 1.35–3.27). A 50-75-year sensitivity cohort produced a similar estimate. Event counts were too small to estimate annual trends or to compare incidence reliably across race and ethnicity groups. Future studies linking EHR data to endoscopy reports, pathology, lesion location, resection technique, margin treatment, surveillance completion, and care outside the index health system are needed to identify preventable CRC mechanisms after EMR and to guide equitable surveillance strategies.

## Data Availability

The data used in this study were obtained from the TriNetX U.S. Collaborative Network. Access to these data is subject to TriNetX data use agreements and cannot be publicly shared. Researchers seeking access should apply directly through the TriNetX platform (https://www.trinetx.com).
